# Editorial: Functional genomics in fruit trees: from ‘omics to sustainable biotechnologies, volume II

**DOI:** 10.3389/fpls.2023.1188832

**Published:** 2023-04-19

**Authors:** Concetta Licciardello, Irene Perrone, Giorgio Gambino, Riccardo Velasco, Manuel Talón

**Affiliations:** ^1^ Research Center for Olive Fruit and Citrus Crops, Council for Agricultural Research and Economics, Acireale, Italy; ^2^ Institute for Sustainable Plant Protection, National Research Council (CNR), Torino, Italy; ^3^ Research Center for Viticulture and Enology, Council for Agricultural Research and Economics, Conegliano, Italy; ^4^ Departamento de Genomica, Instituto Valenciano de Investigaciones Agrarias, Valencia, Spain

**Keywords:** gene-editing, seedlessness, palatability, stomatal density, plant architecture, database, huanglongbing, fruit trees

The Research Topic collects 10 manuscripts focused on cutting-edge topics in functional genomics of fruit trees, that can be grouped into three main themes: advancements in genome editing technologies, availability of tools and approaches for understanding gene family structure and function, and study of gene regulation through long non-coding RNA (lncRNA), transgenesis, and transcriptomic analysis (**Figure 1**). The first set of manuscripts showcases perspectives and limitations of transgene-free genome editing in fruit trees, the use of CRISPR/Cas9 constructs for the reduction of stomatal density in grapevine, and the generation of edited citrus varieties enriched in antioxidant compounds. A second group describes the use of comparative gene family analysis tools, of novel workflows for the Rosaceae, the creation of a comprehensive platform for germplasm innovation and functional genomics in Macadamia, and the construction of a high-density genetic linkage map to identify genetic loci responsible for seedlessness in mandarin. The third set of articles includes studies on citrus focused on the role of lncRNA in response to Huanglongbing (HLB), the fruit-specific expression of Ruby to improve anthocyanin accumulation, and the exploitation of the transcriptome relating to growth and palatability.

**Figure 1:**
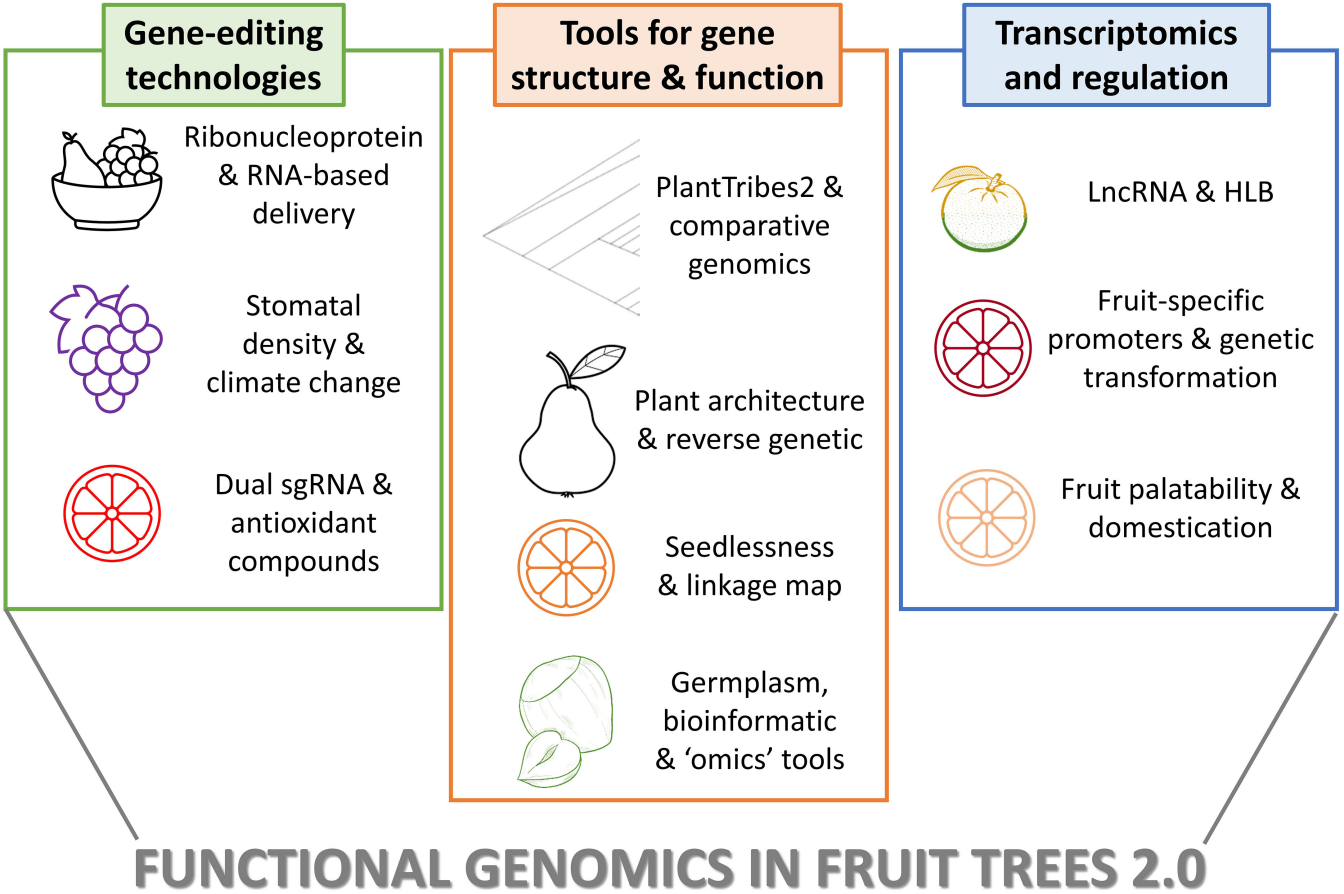
Methods and applications showed in the Editorial Functional Genomics in fruits trees 2.0. The figure summarizes the main approaches reported in the 10 papers collected in Research Topic, organized in the three main groups as summerized in the Editorial. The plant species and the applications are briefly reported.

The recent advancements of new gene-editing tools opens new opportunities for functional studies in fruit trees. Gouthu et al. report the use of a transgene-free gene editing *via* Ribonucleoprotein (RNP) delivery and the ectopic application of RNA-based products; these approaches are mainly addressed to a sustainable and an eco-friendlier environment for a crop production system that could potentially replace the use of chemicals. Both technologies are strictly dependent on the foundational knowledge of gene-to-trait relationships, and the potential and limitations are carefully reviewed.

Through a genome editing approach based on CRISPR/Cas9 technology, Clemens et al. showed the potential of manipulating stomatal density for optimizing grapevine adaptation under changing climate conditions. By inactivation of the *VvEPFL9-1*, a positive regulator of stomata formation, different edited lines of the table grape variety ‘Sugraone’ with a significant reduction in stomatal density and a significant increase in pore length were produced. Interestingly, epfl9-1 mutants showed an improved intrinsic water-use efficiency, a desirable trait to improve plant water conservation and to delay early sugar accumulation.

Salonia et al. used a dual sgRNA approach to knockout the fruit-specific *β-LCY2* to introduce lycopene in five different Tarocco and Sanguigno sweet orange varietal groups. The approach revealed to be highly efficient in introducing point or short mutations, large deletions and the inversion of the region between the cutting site of both sgRNAs. No altered phenotype in vegetative tissues of edited plants has been observed. This work represents the first example of the use of a genome editing approach to potentially improve qualitative traits of citrus fruit.

In an effort to address accessibility and computational challenges in genome-scale research and to rely on comparative genomic approaches that integrate across plant community resources and data types, Wafula et al. provided a valuable tool for the research community working on plant genomics. PlantTribes2 is a scalable, easily accessible, highly customizable, and broadly applicable bioinformatic framework useful for comparative and evolutionary analyses of gene families from any type of organism, including fungi, microbes, animals, and plants. Examples of application are the evaluation of targeted gene family assembly and genome quality. Such as example, Zhang et al. showed an application of PlantTribes2 making simpler the acquisition and the analysis of genome-scale data, through an iterative processes of reverse genetics aimed to understand pear architecture genes. To individuate putative architecture genes in pear, it could be possible to start with genes of interest and the workflow proposed provides a comparative genome approach to efficiently identify, investigate, and then improve and/or validate genes of interest across genomes and genome resources.

Macadamia is an important nut crop, but it’s becoming difficult for researchers to process and use the vast amount of genomic data available. As a central portal, Wang et al. have developed MacadamiaGGD, a database integrating data from germplasm, genomes, transcriptomes, genetic linkage maps, and SSR markers. The database is freely available online and includes bioinformatic tools to conveniently analyze data of interest. The database is expected to broaden the understanding of the germplasm, genetics, and genomics of macadamia species and facilitate molecular breeding efforts.

In citrus, Kumar et al. identified two closely associated SNPs, AX-160417325 and AX-160536283, in Fs-locus on LG5 of ‘Mukaku Kishu’ mandarin. These SNPs reduced the population size and positively predicted seedlessness in 25.0-91.9% of the progenies in studied populations. These markers should be strategic in reducing the effective population size at seedling stage in crosses involving ‘MK’ paternity. Further work will be done, but the availability of these SNPs opens the way in the production of seedless citrus fruits, highly appreciated by consumers.

LncRNAs serve as crucial regulators in plant response to various diseases. Zhuo et al. identified and characterized 8,742 lncRNAs among HLB-tolerant rough lemon and HLB-sensitive sweet orange. LNC_28805 was identified as one of the most important candidate lncRNAs; on the other hands, WRKY33 and SYP121 are two candidate genes targeted by miRNA5021 developing a key role in the bacteria pathogen responses based on the prediction of protein-protein interaction network. This study will be useful in understanding the role of lncRNAs involved in citrus HLB regulation and opens the road for further investigation of their regulatory functions.

Tissue specific promoters are important tools for the precise genetic engineering of crops. Thilmony et al., in the framework of four fruit-preferential promoters, found that CitWax exhibited high fruit-preferential expression of Ruby in Mexican lime. In some of the transgenic trees with high levels of flower and fruit anthocyanin accumulation, leaves deeply coloured at juvenile phase, lost the coloration at maturity. CitWax promoter could control the expression of Ruby increasing the nutritional value and health benefits of citrus fruit.

Pérez-Roman et al. analyzed the transcriptomes of developing fruitlets of wild and domesticated citrus to identify key traits brought about by domestication. Domestication promoted growth processes at the expense of chemical defenses, also impacting in nitrogen and carbon allocation, presumably leading to major differences in organoleptic properties. The production of unpleasant secondary metabolites and acidity, for instance, decreased considerably improving palatability. The results also appear to suggest that domesticated mandarins evolved through progressive refining of other relevant palatability properties.

## Author contributions

All authors drafted the manuscript, revised and approved the final version.

